# 8,11,24-Trioxa-21-thia-19-aza­penta­cyclo­[16.6.0.0^2,7^.0^12,17^.0^19,23^]tetra­cosa-2(7),3,5,12,14,16-hexa­ene

**DOI:** 10.1107/S1600536813012798

**Published:** 2013-05-15

**Authors:** Seenivasan Karthiga Devi, Thothadri Srinivasan, Santhanagopalan Purushothaman, Raghavachary Raghunathan, Devadasan Velmurugan

**Affiliations:** a Centre of Advanced Study in Crystallography and Biophysics, University of Madras, Guindy Campus, Chennai 600 025, India; b Department of Organic Chemistry, University of Madras, Guindy Campus, Chennai 600 025, India

## Abstract

In the title compound, C_19_H_19_NO_3_S, the thia­zole and oxazolidine rings each adopt an envelope conformation, with the S and O atoms as the respective flap atoms. The thia­zole and oxazolidine rings (all atoms) make a dihedral angle of 66.39 (11)° while the phenyl rings subtend a dihedral angle of 22.71 (10)°.

## Related literature

For the biological activity of thia­zole derivatives, see: Guo *et al.* (2006 ▶); Karegoudar *et al.* (2008 ▶); Reddy *et al.* (1999 ▶).
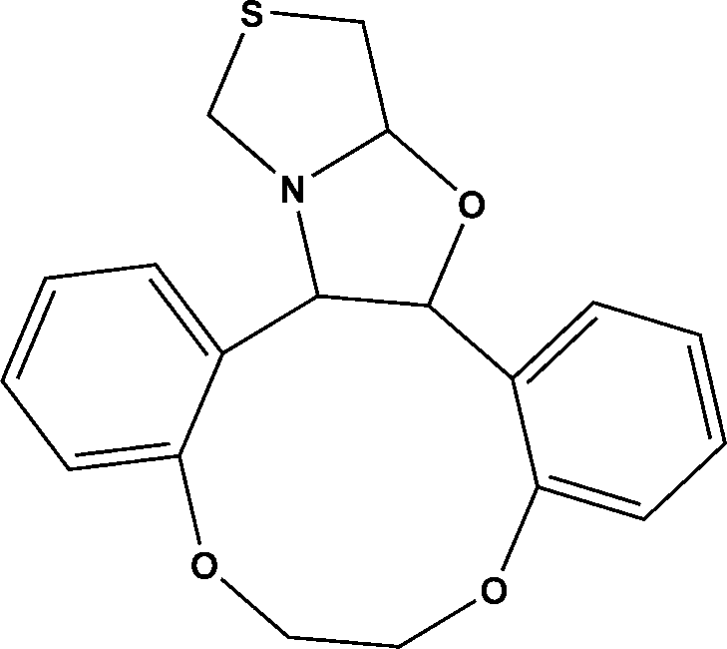


## Experimental

### 

#### Crystal data

C_19_H_19_NO_3_S
*M* *_r_* = 341.42
Monoclinic, 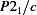

*a* = 10.725 (5) Å
*b* = 10.405 (5) Å
*c* = 14.930 (5) Å
β = 100.262 (5)°
*V* = 1639.4 (12) Å^3^
*Z* = 4
Mo *K*α radiation
μ = 0.22 mm^−1^
*T* = 293 K
0.30 × 0.25 × 0.20 mm

#### Data collection

Bruker SMART APEXII area-detector diffractometer
Absorption correction: multi-scan (*SADABS*; Bruker, 2008 ▶) *T*_min_ = 0.938, *T*_max_ = 0.958
15331 measured reflections
4067 independent reflections
2586 reflections with *I* > 2σ(*I*)
*R*_int_ = 0.030

#### Refinement

*R*[*F* ^2^ > 2σ(*F* ^2^)] = 0.048
*wR*(*F* ^2^) = 0.149
*S* = 1.03
4067 reflections
217 parameters
H-atom parameters constrained
Δρ_max_ = 0.64 e Å^−3^
Δρ_min_ = −0.34 e Å^−3^

### 

Data collection: *APEX2* (Bruker, 2008 ▶); cell refinement: *SAINT* (Bruker, 2008 ▶); data reduction: *SAINT*; program(s) used to solve structure: *SHELXS97* (Sheldrick, 2008 ▶); program(s) used to refine structure: *SHELXL97* (Sheldrick, 2008 ▶); molecular graphics: *ORTEP-3 for Windows* (Farrugia, 2012 ▶); software used to prepare material for publication: *SHELXL97* and *PLATON* (Spek, 2009 ▶).

## Supplementary Material

Crystal structure: contains datablock(s) global, I. DOI: 10.1107/S1600536813012798/bt6902sup1.cif

Structure factors: contains datablock(s) I. DOI: 10.1107/S1600536813012798/bt6902Isup2.hkl

Supplementary material file. DOI: 10.1107/S1600536813012798/bt6902Isup3.cml

Additional supplementary materials:  crystallographic information; 3D view; checkCIF report

## Figures and Tables

**Fig. 1.**
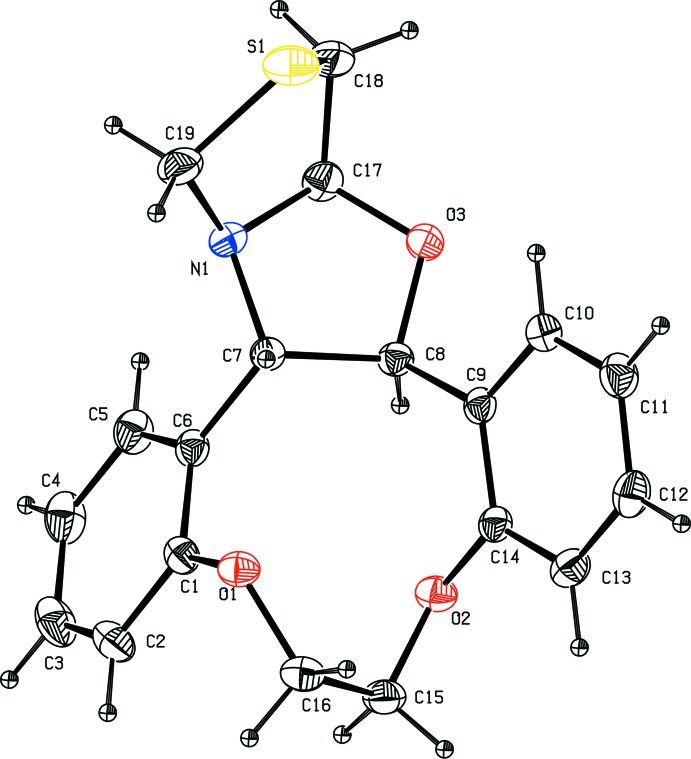
The molecular structure of the title compound, showing displacement ellipsoids drawn at the 30% probability level. H atoms are presented as a small spheres of arbitrary radius.

## supplementary crystallographic information

### Comment

Thiazole derivatives have a varity of physiological effects, such as antiinflammatory (Guo *et al.*, 2006) and antimicrobial (Karegoudar *et al.*, 2008). Against this background, we report herein the crystal structure of the title compound.

In the title compound, C_19_H_19_NO_3_S, (Fig. 1) both the thiazole ring and the oxazolidine ring adopt an *envelope* conformation. The thiazole ring (S1/N1/C17/C18/C19) makes a dihedral angle of 66.39 (11)° with the oxazolidine ring (O3/N1/C7/C8/C17). The thiazole ring makes a dihedral angle of 61.25 (11)° with the phenyl ring (C1-C6), it makes a dihedral angle of 79.60 (11)° with the other phenyl ring (C9-C14).

The oxazolidine ring makes a dihedral angle of 64.80 (11)° with the phenyl ring (C1-C6), it makes a dihedral angle of 67.26 (10)° with the other phenyl ring (C9-C14). The dihedral angle between the two phenyl rings is 22.71 (10)°. The molecular structure features weak intramolecular C–H···O and C–H···N hydrogen bonds (Table 1).

### Experimental

A mixture of 2,2'-(ethane-1,2-diylbis(oxy))dibenzaldehyde (1 mMol) and thiazolidine-4-carboxylic acid (1 mMol) was refluxed in acetonitrile (30ml) for about 5 hrs under N_2_ atm. After the completion of reaction as indicated by TLC, acetonitrile was evaporated under reduced pressure. The crude product was purified by column chromatography using hexane: EtOAc (8:2) mixture as eluent. Single crystals suitable for X-ray diffraction were obtained by slow evaporation of a solution of the title compound in ethyl acetate at room temperature.

### Refinement

The hydrogen atoms were placed in calculated positions and treated as riding atoms: C—H = 0.93 Å to 0.98 Å, with U_iso_(H) = 1.5U_eq_(C) for methyl H atoms and = 1.2U_eq_(C) for other H atoms.

### Crystal data

**Table:**

C_19_H_19_NO_3_S	*F*(000) = 720
*M**_r_* = 341.42	*D*_x_ = 1.383 Mg m^−^^3^
Monoclinic, *P*2_1_/*c*	Mo *K*α radiation, λ = 0.71073 Å
Hall symbol: -P 2ybc	Cell parameters from 4067 reflections
*a* = 10.725 (5) Å	θ = 1.9–28.4°
*b* = 10.405 (5) Å	µ = 0.22 mm^−^^1^
*c* = 14.930 (5) Å	*T* = 293 K
β = 100.262 (5)°	Block, colourless
*V* = 1639.4 (12) Å^3^	0.30 × 0.25 × 0.20 mm
*Z* = 4	

### Data collection

**Table:**

Bruker SMART APEXII area-detector diffractometer	4067 independent reflections
Radiation source: fine-focus sealed tube	2586 reflections with *I* > 2σ(*I*)
Graphite monochromator	*R*_int_ = 0.030
ω and φ scans	θ_max_ = 28.4°, θ_min_ = 1.9°
Absorption correction: multi-scan (*SADABS*; Bruker, 2008)	*h* = −14→14
*T*_min_ = 0.938, *T*_max_ = 0.958	*k* = −13→13
15331 measured reflections	*l* = −19→19

### Refinement

**Table:**

Refinement on *F*^2^	Primary atom site location: structure-invariant direct methods
Least-squares matrix: full	Secondary atom site location: difference Fourier map
*R*[*F*^2^ > 2σ(*F*^2^)] = 0.048	Hydrogen site location: inferred from neighbouring sites
*wR*(*F*^2^) = 0.149	H-atom parameters constrained
*S* = 1.03	*w* = 1/[σ^2^(*F*_o_^2^) + (0.0712*P*)^2^ + 0.3653*P*] where *P* = (*F*_o_^2^ + 2*F*_c_^2^)/3
4067 reflections	(Δ/σ)_max_ < 0.001
217 parameters	Δρ_max_ = 0.64 e Å^−^^3^
0 restraints	Δρ_min_ = −0.34 e Å^−^^3^

### Special details

**Table:**

Geometry. All esds (except the esd in the dihedral angle between two l.s. planes) are estimated using the full covariance matrix. The cell esds are taken into account individually in the estimation of esds in distances, angles and torsion angles; correlations between esds in cell parameters are only used when they are defined by crystal symmetry. An approximate (isotropic) treatment of cell esds is used for estimating esds involving l.s. planes.
Refinement. Refinement of F^2^ against ALL reflections. The weighted R-factor wR and goodness of fit S are based on F^2^, conventional R-factors R are based on F, with F set to zero for negative F^2^. The threshold expression of F^2^ > 2sigma(F^2^) is used only for calculating R-factors(gt) etc. and is not relevant to the choice of reflections for refinement. R-factors based on F^2^ are statistically about twice as large as those based on F, and R- factors based on ALL data will be even larger.

### Fractional atomic coordinates and isotropic or equivalent isotropic displacement parameters (Å^2^)

**Table:**

	*x*	*y*	*z*	*U*_iso_*/*U*_eq_	
C1	0.67608 (18)	0.00360 (19)	0.35262 (13)	0.0435 (4)	
C2	0.5857 (2)	0.0394 (2)	0.40363 (16)	0.0580 (6)	
H2	0.5126	−0.0095	0.4013	0.070*	
C3	0.6040 (3)	0.1464 (3)	0.45719 (16)	0.0685 (7)	
H3	0.5437	0.1692	0.4920	0.082*	
C4	0.7105 (3)	0.2208 (2)	0.46029 (16)	0.0661 (7)	
H4	0.7221	0.2943	0.4963	0.079*	
C5	0.8005 (2)	0.1848 (2)	0.40896 (14)	0.0556 (5)	
H5	0.8725	0.2352	0.4109	0.067*	
C6	0.78602 (17)	0.07568 (18)	0.35488 (12)	0.0417 (4)	
C7	0.88455 (17)	0.03869 (17)	0.29832 (12)	0.0386 (4)	
H7	0.8874	−0.0551	0.2933	0.046*	
C8	0.85966 (16)	0.09868 (18)	0.20073 (12)	0.0404 (4)	
H8	0.8024	0.1723	0.1992	0.048*	
C9	0.80786 (17)	0.00603 (18)	0.12575 (12)	0.0401 (4)	
C10	0.8866 (2)	−0.0566 (2)	0.07553 (13)	0.0487 (5)	
H10	0.9731	−0.0392	0.0872	0.058*	
C11	0.8391 (2)	−0.1444 (2)	0.00832 (14)	0.0587 (6)	
H11	0.8933	−0.1864	−0.0243	0.070*	
C12	0.7101 (2)	−0.1691 (2)	−0.00986 (14)	0.0582 (6)	
H12	0.6774	−0.2282	−0.0547	0.070*	
C13	0.6300 (2)	−0.1062 (2)	0.03824 (14)	0.0517 (5)	
H13	0.5434	−0.1224	0.0255	0.062*	
C14	0.67837 (17)	−0.01947 (18)	0.10528 (12)	0.0404 (4)	
C15	0.50032 (19)	−0.0129 (2)	0.18360 (16)	0.0538 (5)	
H15A	0.4389	−0.0404	0.1314	0.065*	
H15B	0.4584	0.0468	0.2184	0.065*	
C16	0.54456 (18)	−0.1283 (2)	0.24204 (15)	0.0535 (5)	
H16A	0.4819	−0.1488	0.2793	0.064*	
H16B	0.5516	−0.2015	0.2031	0.064*	
C17	1.05652 (19)	0.1675 (2)	0.27269 (14)	0.0507 (5)	
H17	1.0490	0.2583	0.2885	0.061*	
C18	1.1925 (2)	0.1351 (3)	0.27092 (18)	0.0690 (7)	
H18A	1.2486	0.1820	0.3178	0.083*	
H18B	1.2140	0.1560	0.2122	0.083*	
C19	1.1041 (2)	−0.0106 (2)	0.37495 (17)	0.0653 (6)	
H19A	1.0625	−0.0903	0.3861	0.078*	
H19B	1.1540	0.0186	0.4320	0.078*	
N1	1.01005 (14)	0.08607 (16)	0.33910 (11)	0.0472 (4)	
O1	0.66449 (12)	−0.10640 (12)	0.29984 (9)	0.0473 (3)	
O2	0.60208 (12)	0.05173 (13)	0.15245 (9)	0.0488 (4)	
O3	0.98101 (12)	0.14160 (14)	0.18684 (9)	0.0507 (4)	
S1	1.20505 (6)	−0.03571 (7)	0.29167 (6)	0.0782 (3)	

### Atomic displacement parameters (Å^2^)

**Table:**

	*U*^11^	*U*^22^	*U*^33^	*U*^12^	*U*^13^	*U*^23^
C1	0.0465 (10)	0.0417 (10)	0.0441 (10)	0.0101 (8)	0.0126 (8)	0.0106 (8)
C2	0.0566 (13)	0.0594 (14)	0.0645 (13)	0.0105 (10)	0.0288 (11)	0.0127 (11)
C3	0.0815 (18)	0.0714 (16)	0.0609 (14)	0.0299 (14)	0.0351 (13)	0.0091 (12)
C4	0.0878 (18)	0.0574 (14)	0.0537 (12)	0.0204 (13)	0.0147 (12)	−0.0083 (11)
C5	0.0636 (13)	0.0524 (13)	0.0502 (11)	0.0061 (10)	0.0086 (10)	−0.0087 (10)
C6	0.0450 (10)	0.0420 (10)	0.0374 (9)	0.0067 (8)	0.0053 (8)	0.0034 (8)
C7	0.0368 (9)	0.0384 (10)	0.0402 (9)	0.0004 (7)	0.0064 (7)	−0.0012 (8)
C8	0.0376 (9)	0.0396 (10)	0.0436 (9)	−0.0009 (7)	0.0061 (7)	0.0032 (8)
C9	0.0437 (10)	0.0402 (10)	0.0353 (8)	0.0010 (8)	0.0045 (8)	0.0072 (8)
C10	0.0463 (11)	0.0575 (13)	0.0429 (10)	0.0006 (9)	0.0090 (8)	0.0003 (9)
C11	0.0670 (14)	0.0629 (14)	0.0479 (11)	0.0057 (11)	0.0153 (10)	−0.0063 (10)
C12	0.0724 (15)	0.0548 (13)	0.0444 (11)	−0.0044 (11)	0.0025 (10)	−0.0077 (10)
C13	0.0488 (11)	0.0515 (12)	0.0514 (11)	−0.0051 (9)	−0.0007 (9)	0.0001 (10)
C14	0.0410 (10)	0.0410 (10)	0.0379 (9)	0.0019 (8)	0.0036 (8)	0.0063 (8)
C15	0.0374 (10)	0.0616 (13)	0.0622 (12)	0.0019 (9)	0.0083 (9)	0.0054 (11)
C16	0.0416 (11)	0.0523 (13)	0.0676 (13)	−0.0057 (9)	0.0127 (10)	0.0010 (10)
C17	0.0539 (12)	0.0419 (11)	0.0545 (11)	−0.0115 (9)	0.0044 (9)	−0.0019 (9)
C18	0.0471 (12)	0.0852 (18)	0.0733 (15)	−0.0232 (12)	0.0072 (11)	0.0086 (14)
C19	0.0432 (11)	0.0781 (16)	0.0700 (14)	0.0011 (11)	−0.0025 (10)	0.0228 (13)
N1	0.0386 (8)	0.0540 (10)	0.0465 (9)	−0.0036 (7)	0.0012 (7)	−0.0003 (8)
O1	0.0407 (7)	0.0416 (8)	0.0598 (8)	0.0031 (6)	0.0095 (6)	0.0025 (6)
O2	0.0420 (7)	0.0461 (8)	0.0591 (8)	0.0028 (6)	0.0114 (6)	0.0029 (6)
O3	0.0468 (8)	0.0582 (9)	0.0470 (7)	−0.0132 (6)	0.0076 (6)	0.0059 (7)
S1	0.0494 (4)	0.0816 (5)	0.1040 (6)	0.0119 (3)	0.0148 (3)	−0.0004 (4)

### Geometric parameters (Å, º)

**Table:**

C1—O1	1.383 (2)	C12—C13	1.378 (3)
C1—C2	1.386 (3)	C12—H12	0.9300
C1—C6	1.393 (3)	C13—C14	1.379 (3)
C2—C3	1.364 (3)	C13—H13	0.9300
C2—H2	0.9300	C14—O2	1.386 (2)
C3—C4	1.374 (4)	C15—O2	1.428 (2)
C3—H3	0.9300	C15—C16	1.510 (3)
C4—C5	1.386 (3)	C15—H15A	0.9700
C4—H4	0.9300	C15—H15B	0.9700
C5—C6	1.386 (3)	C16—O1	1.434 (2)
C5—H5	0.9300	C16—H16A	0.9700
C6—C7	1.515 (3)	C16—H16B	0.9700
C7—N1	1.461 (2)	C17—O3	1.415 (2)
C7—C8	1.564 (2)	C17—N1	1.458 (3)
C7—H7	0.9800	C17—C18	1.501 (3)
C8—O3	1.426 (2)	C17—H17	0.9800
C8—C9	1.506 (3)	C18—S1	1.805 (3)
C8—H8	0.9800	C18—H18A	0.9700
C9—C10	1.388 (3)	C18—H18B	0.9700
C9—C14	1.393 (3)	C19—N1	1.457 (3)
C10—C11	1.386 (3)	C19—S1	1.808 (3)
C10—H10	0.9300	C19—H19A	0.9700
C11—C12	1.386 (3)	C19—H19B	0.9700
C11—H11	0.9300		
			
O1—C1—C2	122.33 (19)	C12—C13—H13	120.0
O1—C1—C6	116.70 (16)	C14—C13—H13	120.0
C2—C1—C6	120.9 (2)	C13—C14—O2	122.64 (17)
C3—C2—C1	120.0 (2)	C13—C14—C9	121.01 (18)
C3—C2—H2	120.0	O2—C14—C9	116.27 (16)
C1—C2—H2	120.0	O2—C15—C16	112.49 (16)
C2—C3—C4	120.8 (2)	O2—C15—H15A	109.1
C2—C3—H3	119.6	C16—C15—H15A	109.1
C4—C3—H3	119.6	O2—C15—H15B	109.1
C3—C4—C5	119.0 (2)	C16—C15—H15B	109.1
C3—C4—H4	120.5	H15A—C15—H15B	107.8
C5—C4—H4	120.5	O1—C16—C15	112.16 (16)
C4—C5—C6	121.8 (2)	O1—C16—H16A	109.2
C4—C5—H5	119.1	C15—C16—H16A	109.2
C6—C5—H5	119.1	O1—C16—H16B	109.2
C5—C6—C1	117.49 (18)	C15—C16—H16B	109.2
C5—C6—C7	121.07 (18)	H16A—C16—H16B	107.9
C1—C6—C7	121.41 (17)	O3—C17—N1	107.18 (15)
N1—C7—C6	111.36 (15)	O3—C17—C18	110.00 (18)
N1—C7—C8	104.20 (14)	N1—C17—C18	109.38 (18)
C6—C7—C8	113.43 (14)	O3—C17—H17	110.1
N1—C7—H7	109.2	N1—C17—H17	110.1
C6—C7—H7	109.2	C18—C17—H17	110.1
C8—C7—H7	109.2	C17—C18—S1	105.17 (14)
O3—C8—C9	108.52 (14)	C17—C18—H18A	110.7
O3—C8—C7	104.59 (14)	S1—C18—H18A	110.7
C9—C8—C7	114.59 (15)	C17—C18—H18B	110.7
O3—C8—H8	109.7	S1—C18—H18B	110.7
C9—C8—H8	109.7	H18A—C18—H18B	108.8
C7—C8—H8	109.7	N1—C19—S1	107.90 (15)
C10—C9—C14	118.11 (18)	N1—C19—H19A	110.1
C10—C9—C8	121.49 (17)	S1—C19—H19A	110.1
C14—C9—C8	120.40 (16)	N1—C19—H19B	110.1
C11—C10—C9	121.33 (19)	S1—C19—H19B	110.1
C11—C10—H10	119.3	H19A—C19—H19B	108.4
C9—C10—H10	119.3	C17—N1—C19	110.68 (17)
C12—C11—C10	119.3 (2)	C17—N1—C7	108.30 (15)
C12—C11—H11	120.3	C19—N1—C7	116.49 (17)
C10—C11—H11	120.3	C1—O1—C16	117.03 (14)
C13—C12—C11	120.2 (2)	C14—O2—C15	117.95 (16)
C13—C12—H12	119.9	C17—O3—C8	108.54 (14)
C11—C12—H12	119.9	C18—S1—C19	86.56 (12)
C12—C13—C14	120.0 (2)		
			
O1—C1—C2—C3	177.59 (19)	C10—C9—C14—C13	1.2 (3)
C6—C1—C2—C3	−0.2 (3)	C8—C9—C14—C13	−178.50 (17)
C1—C2—C3—C4	1.0 (3)	C10—C9—C14—O2	−175.54 (16)
C2—C3—C4—C5	−0.8 (4)	C8—C9—C14—O2	4.7 (2)
C3—C4—C5—C6	−0.2 (3)	O2—C15—C16—O1	37.5 (3)
C4—C5—C6—C1	1.0 (3)	O3—C17—C18—S1	−83.13 (18)
C4—C5—C6—C7	179.23 (18)	N1—C17—C18—S1	34.3 (2)
O1—C1—C6—C5	−178.67 (16)	O3—C17—N1—C19	112.63 (19)
C2—C1—C6—C5	−0.8 (3)	C18—C17—N1—C19	−6.6 (2)
O1—C1—C6—C7	3.1 (2)	O3—C17—N1—C7	−16.2 (2)
C2—C1—C6—C7	−179.04 (17)	C18—C17—N1—C7	−135.44 (18)
C5—C6—C7—N1	27.6 (2)	S1—C19—N1—C17	−24.3 (2)
C1—C6—C7—N1	−154.15 (16)	S1—C19—N1—C7	100.01 (18)
C5—C6—C7—C8	−89.5 (2)	C6—C7—N1—C17	−122.40 (17)
C1—C6—C7—C8	88.7 (2)	C8—C7—N1—C17	0.25 (19)
N1—C7—C8—O3	15.47 (18)	C6—C7—N1—C19	112.12 (19)
C6—C7—C8—O3	136.75 (16)	C8—C7—N1—C19	−125.24 (18)
N1—C7—C8—C9	134.16 (16)	C2—C1—O1—C16	47.3 (2)
C6—C7—C8—C9	−104.56 (18)	C6—C1—O1—C16	−134.80 (18)
O3—C8—C9—C10	20.6 (2)	C15—C16—O1—C1	51.5 (2)
C7—C8—C9—C10	−95.9 (2)	C13—C14—O2—C15	44.0 (2)
O3—C8—C9—C14	−159.70 (16)	C9—C14—O2—C15	−139.28 (17)
C7—C8—C9—C14	83.8 (2)	C16—C15—O2—C14	55.3 (2)
C14—C9—C10—C11	−1.5 (3)	N1—C17—O3—C8	27.1 (2)
C8—C9—C10—C11	178.18 (18)	C18—C17—O3—C8	145.91 (17)
C9—C10—C11—C12	0.8 (3)	C9—C8—O3—C17	−148.93 (15)
C10—C11—C12—C13	0.3 (3)	C7—C8—O3—C17	−26.20 (19)
C11—C12—C13—C14	−0.6 (3)	C17—C18—S1—C19	−40.58 (17)
C12—C13—C14—O2	176.39 (17)	N1—C19—S1—C18	37.84 (17)
C12—C13—C14—C9	−0.2 (3)		

### Hydrogen-bond geometry (Å, º)

**Table:**

*D*—H···*A*	*D*—H	H···*A*	*D*···*A*	*D*—H···*A*
C5—H5···N1	0.93	2.51	2.834 (3)	101
C7—H7···O1	0.98	2.47	2.805 (3)	100
C10—H10···O3	0.93	2.39	2.728 (3)	101
